# APP Osaka Mutation in Familial Alzheimer’s Disease—Its Discovery, Phenotypes, and Mechanism of Recessive Inheritance

**DOI:** 10.3390/ijms21041413

**Published:** 2020-02-19

**Authors:** Takami Tomiyama, Hiroyuki Shimada

**Affiliations:** 1 Department of Translational Neuroscience, Osaka City University Graduate School of Medicine, Osaka 545-8585, Japan; 2 Clinical Research Center for Dementia, Osaka City University Graduate School of Medicine, Osaka 545-8585, Japan; h.shimada@med.osaka-cu.ac.jp

**Keywords:** APP mutation, recessive inheritance, familial Alzheimer’s disease, Aβ oligomers, amyloid imaging

## Abstract

Alzheimer’s disease is believed to begin with synaptic dysfunction caused by soluble Aβ oligomers. When this oligomer hypothesis was proposed in 2002, there was no direct evidence that Aβ oligomers actually disrupt synaptic function to cause cognitive impairment in humans. In patient brains, both soluble and insoluble Aβ species always coexist, and therefore it is difficult to determine which pathologies are caused by Aβ oligomers and which are caused by amyloid fibrils. Thus, no validity of the oligomer hypothesis was available until the Osaka mutation was discovered. This mutation, which was found in a Japanese pedigree of familial Alzheimer’s disease, is the deletion of codon 693 of APP gene, resulting in mutant Aβ lacking the 22nd glutamate. Only homozygous carriers suffer from dementia. In vitro studies revealed that this mutation has a very unique character that accelerates Aβ oligomerization but does not form amyloid fibrils. Model mice expressing this mutation demonstrated that all pathologies of Alzheimer’s disease can be induced by Aβ oligomers alone. In this review, we describe the story behind the discovery of the Osaka mutation, summarize the mutant’s phenotypes, and propose a mechanism of its recessive inheritance.

## 1. Introduction

Alzheimer’s disease (AD) is believed to begin with synaptic dysfunction caused by soluble Aβ oligomers. This idea, the so-called oligomer hypothesis, was proposed in 2002 [1] and has been supported by growing amounts of evidence from animal and organotypic experiments [2,3,4,5,6,7,8]. However, there was no direct evidence that Aβ oligomers actually disrupt synaptic function to cause cognitive impairment in humans. In the brains of AD patients, both soluble and insoluble Aβ species always coexist from the disease onset, with the latter usually observed as senile plaques in the brain parenchyma. The existence of senile plaques, along with neurofibrillary tangles (NFTs) that are composed of hyperphosphorylated tau and brain atrophy that comes from neuron loss, is an absolute requirement for the diagnosis of AD. In such circumstances, the determination of the pathological roles of Aβ oligomers separately from those of amyloid fibrils is nearly impossible, and therefore no validity of the oligomer hypothesis was available. To prove this hypothesis in humans, we needed clinical cases that developed AD without forming senile plaques, even though the feature of no senile plaques is contradictory to the definition of AD. Aside from this discrepancy, if we could find such patients and if we could identify novel etiologic mutation(s) in those patients, we could create new disease models to investigate the pathological and physiological roles of Aβ oligomers at the molecular, cellular, and individual levels for basic and translational research. However, we never expected such clinical cases to exist.

The Arctic mutation (E693G) in amyloid precursor protein (APP), which was discovered in a Swedish family showing clinical symptoms of early-onset AD [9], has been shown to accelerate Aβ protofibril formation in vitro, a soluble intermediate oligomer of Aβ fibrillization, and intraneuronal Aβ aggregation in model mice [10,11]. In those mice, intracellular Aβ aggregation occurred concomitantly with cognitive impairment before the onset of amyloid plaques. These features imply that the Arctic mutation might be favorable to study the pathological effects of Aβ oligomers, but the patients displayed AD pathologies including severe congophilic angiopathy, region-specific NFTs, and abundant amyloid plaques [12,13]. Interestingly, those plaques had a characteristic ring-like architecture and were negative for Congo red staining [12,13] with a low retention in Pittsburgh compound-B (PIB)-PET scans [14].

Meanwhile, in 2001, we had an opportunity to come across a very unusual case of hereditary dementia. The proband (female) showed typical clinical symptoms of AD, but almost no signals of senile plaques in PIB-PET scans [15]. From her pedigree, we identified a novel APP mutation at the same position as the Arctic mutation. This ‘Osaka’ mutation (E693Δ) was found to have a very unique character that accelerates Aβ oligomerization but does not form amyloid fibrils. This was the very mutation we had eagerly sought, but its discovery was quite accidental.

## 2. Discovery of the Osaka Mutation

**Figure 1:**
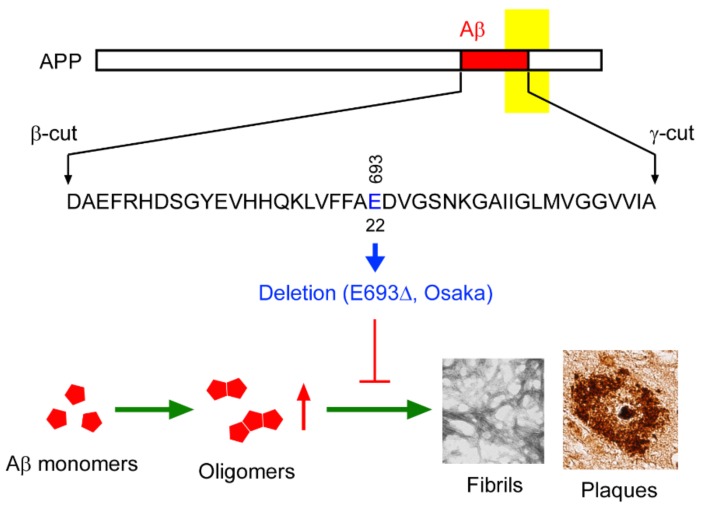
Identity of the Osaka mutation. The Osaka mutation is the deletion of codon 693 of APP gene that produces mutant Aβ lacking the 22nd glutamate. This mutation has a very unique character that accelerates Aβ oligomerization (red arrow) but does not form amyloid fibrils (T-shaped red line). Green arrows indicate Aβ aggregation process.

**Figure 2:**
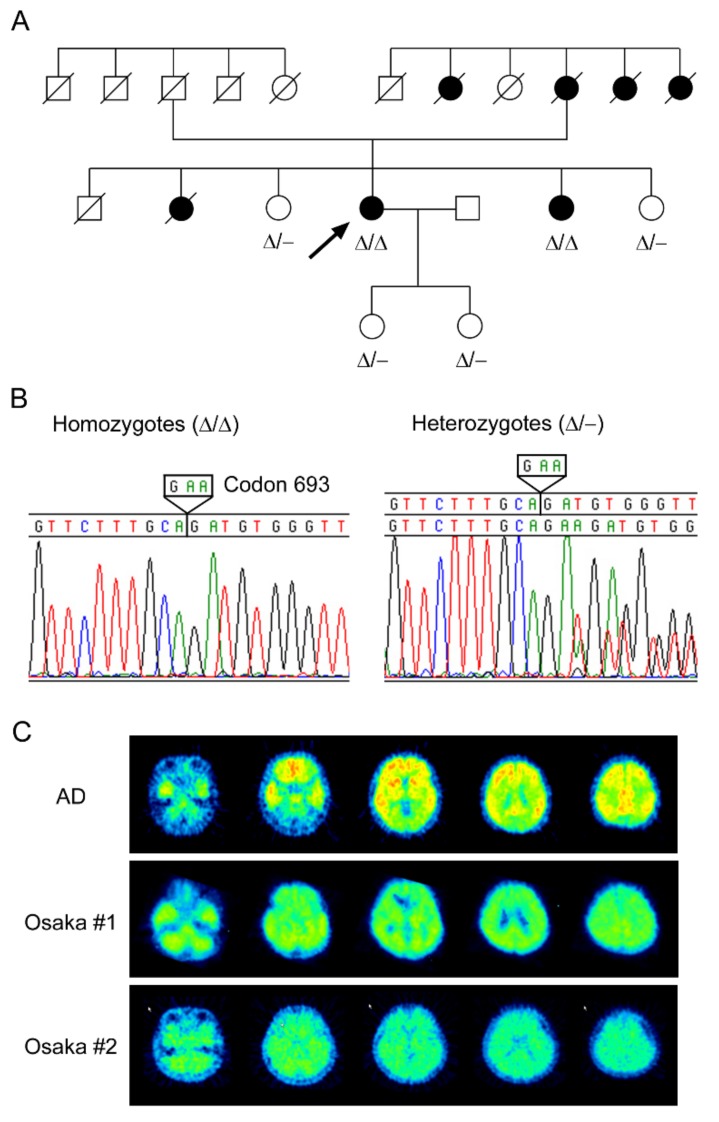
Recessive inheritance of the Osaka mutation. (**A**) Pedigree of familial Alzheimer’s disease (AD) with the Osaka mutation. The proband is indicated with an arrow. Both of the affected members were homozygous (Δ/Δ) for the mutation, while unaffected family members were all heterozygous (Δ/-). Square, male; circle, female. (**B**) DNA sequence of the Osaka mutation. Homozygous and heterozygous deletion of codon 693 of APP gene were detected in the pedigree. (**C**) No amyloid plaques in the Osaka mutation. Pittsburgh compound-B (PIB-PET) images of AD (71-year-old woman), the proband (62-year-old woman), and her affected sister (61-year-old woman) are shown.

In 2001, a Japanese woman who recognized memory disturbance visited the Osaka City University Hospital. She was 57 years old at the time and was diagnosed with mild cognitive impairment (MCI). The doctor in charge (the second author of this review) noticed that her pedigree had many patients with dementia and suspected familial AD. The doctor asked the first author’s group to conduct a genetic test. We examined the patient’s APP, presenilin 1, and presenilin 2 genes, and in September 2002, we discovered a new mutation on APP. This mutation was the first deletion-type mutation found in APP; the codon GAA encoding the 693rd glutamate, which corresponds to the 22nd amino acid in the Aβ sequence, had disappeared [15] (Figure 1). Interestingly, the woman had this mutation in both APP alleles, i.e., she was homozygous (Figure 2A,B). She showed progressive cognitive decline and was diagnosed as having AD at the age of 59. Under informed consent, we carried out gene examination for other family members. One of her younger sisters was found to also have this mutation in both APP alleles, while her elder sister and another younger sister and her two daughters possessed this mutation only in one allele. Their cognitive function was normal at the time, but the homozygous sister soon developed dementia at the same age (59 years old), as did the proband. ApoE genotype was ε3/ε3 for all sisters including the proband, and ε3/ε4 for the two daughters. These findings suggest that the inheritance mode of this mutation is recessive, which was the first case in familial AD.

This mutation, which we named the Osaka mutation, is located within the Aβ sequence, and therefore we considered that this mutation might affect the production and/or aggregation of Aβ like other pathological APP mutations (https://www.alzforum.org/mutations/app). Thus, we initially studied the effects of this mutation on Aβ production in cultured cells. HEK293 cells were transfected with mutant APP, and Aβ secretion was compared with wild-type APP. The ratio of Aβ40 and Aβ42 was almost the same as wild-type, but Aβ secretion was nearly half the level of wild-type [15]. Subsequently, we tested Aβ aggregation and toxicity using synthetic peptides. In PBS solution, neither mutant Aβ40 nor Aβ42 peptides showed any increase in thioflavin T fluorescence, which reflects the level of β-sheet conformation, even after 7 days incubation [15]. The aged peptide solutions were subjected to electron microscopy, and no amyloid fibrils were observed. Under the same condition, wild-type Aβ peptides showed a rapid increase in thioflavin T fluorescence and abundant amyloid fibrils. When added to human and mouse neuronal cells (IMR-32 and Neuro2a, respectively), wild-type, but not mutant Aβ42 peptide, showed toxicity in the MTT assay [16]. Soon after the discovery of the Osaka mutation, we started to generate transgenic (Tg) mice expressing human APP695 with this mutation under the mouse prion protein promoter. In the next year (2003), three mutant lines with different levels of APP expression were established. The expression level of human APP was almost the same as that of endogenous mouse APP in line 1 and was lower in lines 2 and 3. We examined brain sections from adult mice of line 1 for amyloid deposition, but no plaque-like structures were observed [17]. These results, for example, lowered Aβ production and aggregation in vitro and no amyloid plaques in vivo, which appeared to be against our expectation that the Osaka mutation plays an etiological role in AD. Rather, this mutation seemed protective, although the mutant peptides were found more resistant to enzymatic degradation than wild-type peptides [15]. We began to suspect that the Osaka mutation might be just a harmless genetic polymorphism that happened to occur in the pedigree of familial dementia.

In the summer of 2003, when looking at the results of Western blots, we unexpectedly noticed that the mutant Aβ peptide formed more abundant oligomers than wild-type peptide [15,16]. These oligomers (dimers, trimers, and tetramers) appeared to be very stable and did not proceed into larger aggregates of amyloid fibrils. These findings that the mutant Aβ peptides tend to form oligomers but never fibrils seemed very unusual, because we thought that once Aβ starts to aggregate, the process does not stop until all monomers and oligomers are spent for fibril formation. In the same period, we studied the effects of the Osaka mutation on APP processing in transfected cells. This mutation suppressed neither β- nor γ-cut of APP, indicating no inhibition of Aβ production [18]. Nevertheless, Aβ secretion was decreased. Soon after, we found that mutant APP-expressing cells contained more abundant intracellular Aβ than wild-type APP-expressing cells [18]. In addition, the intracellular Aβ was accumulated as monomers, dimers, and perhaps trimers. This finding was later supported by immunostaining of the cells with Aβ oligomer-selective antibodies. If the Osaka mutation really does promote Aβ oligomerization, it could be a cause of AD by exacerbating oligomer-induced synaptic alteration regardless of the lack of amyloid plaques. In fact, when added to mouse hippocampal slice culture, the mutant Aβ42 peptide caused a decrease of synaptophysin, a marker of pre-synapses, more potently than wild-type peptide [16]. This was the first time we realized that the Osaka mutation could prove the oligomer hypothesis in humans.

This notion led us to evaluate the toxic effects of mutant Aβ on synaptic plasticity by in vivo electrophysiology. Synthetic Aβ42 peptide was injected into rat cerebral ventricles at a dose of 10 ng. Ten minutes after the injection, high frequency stimulation (HFS) was delivered to the Schaffer collateral/commissural pathway, and field excitatory postsynaptic potential (fEPSP) was measured in the hippocampal CA1 region. Compared with wild-type Aβ, mutant Aβ suppressed long-term potentiation (LTP) more potently [15]. This result appeared to support our hypothesis that the Osaka mutation aggravates Aβ-induced synaptic dysfunction by accelerating its oligomerization.

From these results, we decided to answer a bigger question: Do patients with the Osaka mutation really have no senile plaques in their brains? In 2006, we commissioned the attending doctor (the second author), who was an expert on amyloid imaging, to perform PIB-PET analysis for the proband who was 62 years old at the time. Only slight signals were detected in the temporal, parietal, and occipital lobes and in the cerebellum, but not in the frontal lobe [15] (Figure 2C). The signals were far less than those in typical AD, indicating that there were almost no senile plaques in the patient. We next examined Aβ levels in her cerebrospinal fluid (CSF). The amounts of Aβ40 and Aβ42 and the ratio of Aβ42/Aβ40 were considerably lower than those in control patients with AD or other neurological disorders, whereas the ratio of Aβ oligomers to monomers was markedly higher than those in control patients [15]. The low levels of Aβ40 and Aβ42 in the CSF presumably reflect their accumulation in the brain parenchyma, and the high ratio of oligomers to monomers confirms the oligomer-prone aggregation property of mutant Aβ in vivo. Based on these results, we concluded that the Osaka mutation causes AD by accelerating the formation of synaptotoxic Aβ oligomers without forming amyloid plaques.

In the fall of 2007, we presented our findings at domestic and international conferences without disclosing the identity of this mutation. Our discovery of an oligomer-prone mutation garnered great interest from the audience (https://www.alzforum.org/news/conference-coverage/san-diego-oligomers-live-bad-reputation-part-2). In addition, our claim that AD develops by Aβ oligomers alone and that senile plaques are not necessary for disease onset was controversial, because it opposed the definition of AD, in that the existence of senile plaques is an absolute requirement for the disease. In the next year, the first report of the Osaka mutation was finally published [15] after it had been rejected by several journals, partly due to the lack of autopsy data and the small number of patients. However, since its publication, the Osaka mutation has been appreciated as the first evidence showing that the mechanism of the oligomer hypothesis actually applies to humans.

## 3. Phenotypes of the Osaka Mutation

### 3.1. Patients

Two pedigrees of familial AD with the Osaka mutation have so far been reported. One is from Osaka City University, Osaka, Japan [15,19], and the other is from Kawasaki Medical School, Okayama, Japan [20]. Probably both pedigrees originated from the same island in the Inland Sea of Japan. Two patients in the first pedigree (Osaka) and three patients in the second pedigree (Okayama) have been described. They were all homozygous for the Osaka mutation, while their unaffected family members were all heterozygous. In the first report of the Osaka mutation, we screened 5310 Japanese people recruited for the Japanese Genetic Study Consortium for AD for this mutation and found three independent additional carriers [15]. One was homozygous and had AD at 36 years old. The other two were heterozygous; one had MCI at 81 years old and the other was cognitively normal at 64 years old. These results clearly indicate that this mutation is recessive. In PIB-PET scans, all patients tested in the two pedigrees were almost negative, supporting our conclusion that this mutation causes disease without forming amyloid plaques.

The proband of the first pedigree experienced memory disturbance at 55 years old [15,19]. Her MMSE scores and MRI and fluorodeoxyglucose (FDG)-PET images were rather normal at 57 years old. However, three-dimensional stereotactic surface projection (3D-SSP) analysis showed a hypometabolism of FDG in the posterior cingulate cortex, which is similar to typical AD. At 59 years old, her MMSE score decreased to 22. According to the Diagnostic and Manual Statistical of Mental Disorders, Third Edition (DSM-IIIR), and the criteria of the National Institute of Neurological and Communication Disorders-Alzheimer’s Disease and Related Disorders Association (NINC DS-ADRDA), she was diagnosed with AD. Two years later, at 61 years old, her MMSE score decreased to 18. She developed cerebellar ataxia, gait disturbance, ideomotor apraxia, and pyramidal signs. These symptoms are unusual in AD. At 62 years old, her MMSE score further decreased to 5. Nevertheless, MRI scans displayed only mild parietal lobe atrophy. Furthermore, [^11^C]PIB-PET scans revealed almost no amyloid accumulation (Figure 2C). FDG-PET scans showed decreased glucose metabolism throughout the brain except the motor and sensory cortices and the cerebellum. At 63 years old, she became unable to walk. At 65 years old, MRI scans revealed moderate atrophy of the hippocampus, mild atrophy of the cerebrum, and mild dilatation of the third and lateral ventricles. The levels of Aβ42 and Aβ40 in her CSF were 4.1 and 242.8 pg/mL, respectively, and those of total tau and phosphorylated tau were 628 and 87.2 pg/mL, respectively. These values alone suggest that she was in an advanced phase of AD. We decided to perform tau imaging analysis for her at 70 years old with the [^11^C]PBB3 probe. PET scans revealed massive NFT formation in the cerebellum and cerebral cortex (Shimada et al. unpublished observations). In addition, MRI scans revealed severe brain atrophy. These observations demonstrate that the pathological cascade of AD proceeds even in the absence of amyloid plaques.

The second patient in the first pedigree, the younger sister of the proband, experienced memory disturbance at 59 years old [19]. Her MMSE score was 27 at the time. Her memory gradually worsened, and at 61 years old, her MMSE score was 15. Similar to the proband, she exhibited cerebellar ataxia, gait disturbance, and pyramidal signs at this age. MRI scans displayed only mild cortical atrophy. PIB-PET scans revealed no amyloid accumulation (Figure 2C). The levels of Aβ42 and Aβ40 in her CSF were 9.5 and 285.2 pg/mL, respectively, and those of total tau and phosphorylated tau were 856 and 152 pg/mL, respectively. Again, these values alone suggest that she was in an advanced phase of AD. At 62 years old, she also became unable to walk.

Taken together, these two patients in the same pedigree developed very similar symptoms and showed a similar progression of the disease. In the early stages, they exhibited only memory disturbance without evident brain atrophy or evident amyloid deposition. In the late stages, they displayed mild to moderate brain atrophy and unexpected motor dysfunction. These clinical features are unique and distinctive from typical AD. To elucidate the underlying mechanism, neuropathological examination by brain autopsy is required for these patients. However, both patients are already deceased at 70 and 66 years old, respectively, and unfortunately, we could not obtain autopsy samples from them.

Compared with the first pedigree, the proband (female) in the second pedigree showed an earlier onset of dementia. According to one report [20], she experienced memory disturbance at 35 years old. She was diagnosed with AD at 42 years old based on progressive cognitive impairment and prominent spatial disorientation. At 48 years old, she had difficulty walking and became bedridden by 50 years old. She had spastic paraparesis and mild dysphagia at 56 years old. The serum level of Aβ and CSF levels of total tau and phosphorylated tau were normal. MRI scans showed remarkable brain atrophy, and FDG-PET scans displayed a greater reduction in glucose uptake in the cerebral cortex compared with typical AD. Nevertheless, PIB-PET scans revealed no amyloid deposition at 56 years old. Using Western blots, the authors detected high-molecular weight Aβ oligomers in her CSF under the non-denaturing condition, while the total level of Aβ under the denaturing condition was less than control level. Her elder brother and sister had memory disturbance at 59 and 44 years old, respectively. They also had difficulty walking due to spasticity of the lower limbs at 66 and 58 years old, respectively. Thus, similar to the first pedigree, the second pedigree also exhibited motor dysfunction, which is unusual in AD. The authors stated that compared with the first pedigree, motor impairment was more profound and brain atrophy was more severe in the second pedigree.

### 3.2. Animal Models

**Figure 3:**
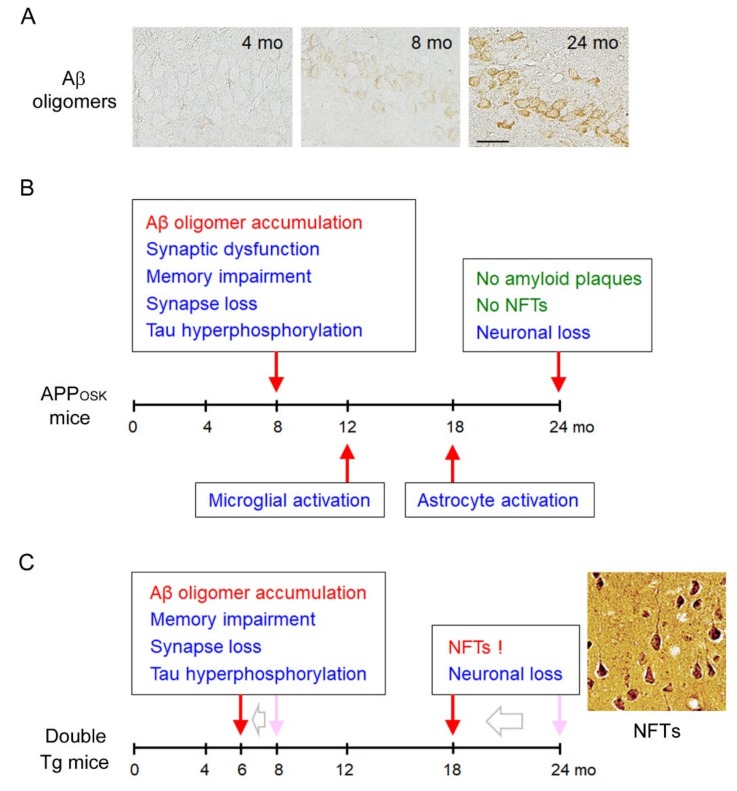
Phenotypes of APP_OSK_ mice. (**A**) Intraneuronal accumulation of Aβ oligomers in APP_OSK_ mice. The mice started to accumulate intracellular Aβ oligomers at 8 months. Images of the hippocampus are shown. Scale bar = 30 μm. (**B**) Aβ oligomers alone can induce most AD pathologies except for neurofibrillary tangles (NFT) formation. (**C**) The double Tg mice expressing both the Osaka mutation and wild-type human tau displayed NFTs at 18 months. Intracellular Aβ oligomers and tau might interact to accelerate each other’s pathologies.

As mentioned above, we established Tg mice expressing Osaka-mutant APP in 2003. This model, which we named APP_OSK_ mice, did not show any amyloid plaques in our first examination. However, because we had learned the unique character of the Osaka mutation, we re-examined brain sections of the mice. Using Aβ oligomer-selective antibodies [21,22], we found that APP_OSK_ mice start to accumulate Aβ oligomers within neurons in the hippocampus, cerebral cortex, and cerebellum at 8 months [17] (Figure 3A). This accumulation was age-dependent, but amyloid plaque deposition was not detected even at 24 months. In accordance with Aβ accumulation, the level of synaptophysin in the hippocampus began to decrease at 8 months. We measured the synaptic plasticity of 8-month-old APP_OSK_ mice by in vivo electrophysiology in comparison with wild-type APP Tg mice (referred to as APP_WT_ mice) [23], which show almost the same level of APP expression as APP_OSK_ mice. HFS was delivered to the perforant path, and population spikes were recorded in the granular cell body layer of the dentate gyrus. Compared with non-Tg mice, basal synaptic transmission was not affected in either APP_WT_ or APP_OSK_ mice [17]. On the other hand, paired-pulse facilitation (PPF), a measure of short-term synaptic plasticity, and LTP were significantly inhibited in both Tg mice, with more severe deterioration in APP_OSK_ mice. Then we examined the cognitive function of APP_OSK_ mice at 8 months by the Morris water maze test. Compared with non-Tg mice, APP_WT_ mice showed only slight impairment in memory, but the memory of APP_OSK_ mice was markedly disturbed [17]. These results were in agreement with the hypothesis that Aβ oligomers cause early synaptic pathology in AD.

In AD brain, not only synaptic alterations but also many other pathologies are induced in parallel with Aβ accumulation, including tau hyperphosphorylation, NFT formation, glial activation, and neuron loss. It had been unclear which pathologies are attributable to Aβ oligomers and which are not. To address this question, we examined brain sections of APP_OSK_ mice for these pathologies [17]. Tau hyperphosphorylation occurred at 8 months in the hippocampal mossy fibers and at 12 months in the cerebral cortex, but NFTs were not observed even at 24 months. Microglial activation was first detected at 12 months, while astrocyte activation was at 18 months in both the hippocampus and cerebral cortex. Finally, neuron loss was observed at 24 months in the pyramidal cell layer of the hippocampal CA3 region, but not in the cerebral cortex at that age. These results clearly indicate that Aβ oligomers alone can induce most AD pathologies except for NFT formation (Figure 3B).

We speculated that NFT formation did not occur in APP_OSK_ mice because the mice express only mouse tau, which is different from human tau in amino acid sequence and isoform expression at adult age. If those mice expressed human tau with the same pattern of isoform expression, NFTs might be formed by the action of Aβ oligomers. Thus, we crossed APP_OSK_ mice with wild-type tau Tg mice. The latter were generated in our laboratory so that they express the longest 3-repeat and 4-repeat human tau isoforms under the mouse calcium/calmodulin-dependent kinase IIa promoter and were found not to show any pathologies, even at 24 months [24]. On the other hand, the double Tg mice displayed NFTs at 18 months in the hippocampus and cerebral cortex [25]. Interestingly, Aβ oligomer accumulation, tau hyperphosphorylation, synapse loss, and memory impairment were all accelerated in double Tg mice beginning at 6 months, and neuron loss was also started at 18 months. These findings demonstrate that Aβ oligomers can initiate the pathological cascade of AD including NFT formation and that intracellular Aβ oligomers and tau presumably interact to accelerate each other’s pathologies (Figure 3C).

Our finding that Tg mice expressing the Osaka mutation exhibit an intraneuronal accumulation of Aβ oligomers but not extracellular deposition of amyloid plaques was supported by another research group. Kulic et al. [26] created a new mouse model that expresses human APP695 with both the Osaka and Swedish (K670N/M671L) mutations under the same mouse prion protein promoter as APP_OSK_ mice. These double mutant Tg mice displayed an intraneuronal accumulation of Aβ oligomers at 3 months, but no amyloid plaque deposition up to 15 months. Instead, the mice exhibited vascular amyloid deposits in the leptomeningeal cerebellar and cortical vessels at 24 months. These deposits were positive for thioflavin S and Congo red staining, indicating the existence of fibrillar aggregates of Aβ. In our APP_OSK_ mice, intracellular Aβ oligomers were only faintly thioflavin S-positive [17]. Nevertheless, they were mostly collected in SDS-insoluble fractions, implying that Osaka-mutant Aβ oligomers may possess insoluble fibrillar nature with the β-sheet conformation, as shown in an in vitro study [27].

Several groups have observed the opposite phenomena to our findings in experiments using synthetic peptides, showing that their own Osaka-mutant peptides promptly aggregated into amyloid fibrils [28,29,30,31,32,33,34,35]. However, we have often experienced that the aggregation property of synthetic peptides show remarkable lot-to-lot variations and that peptide aggregation largely depends on the experimental conditions, including the environment surrounding the peptides. In vitro aggregation studies are usually performed in test tubes only containing the peptides in a pure solution like PBS, which is completely different from the circumstances in the brain. We therefore consider that in vitro results using synthetic peptides are less reliable than in vivo observations in patients and animal models. Our initial in vitro finding that Osaka-mutant peptides form abundant oligomers but no amyloid fibrils have been confirmed in mouse models and patients [15,17,19,20].

There is an interesting animal model to study the pathological roles of Aβ oligomers [36]. A comparison of Aβ sequences of several animal species shows that chimpanzee and dog have the identical sequence as humans, while cat sequence differs by 1 amino acid (D7E) and mice sequence differs by 3 amino acids (R5G, Y10F, and H13R). Chimpanzee and dog display amyloid plaques in their brains, whereas cats accumulate only intraneuronal Aβ oligomers without amyloid plaques, and mice have no amyloid pathology. On the other hand, the amino acid sequence of tau is identical to human only in chimpanzees, and has homologies of 92% in dog, 93% in cat, and 89% in mice. All species but mice express 3-repeat and 4-repeat tau isoforms at an adult age like humans, whereas a mouse expresses only 4-repeat tau isoforms. Nonetheless, NFTs and neurodegeneration were observed only in aged cats. These results suggest that not amyloid plaques, but intracellular Aβ oligomers, can initiate the pathological cascade of AD, leading to NFT formation and neuron death. This finding appears to support our notion that Aβ oligomers can induce NFTs.

### 3.3. Cellular Models

Cell culture system has long been used to investigate pathological and physiological roles of Aβ. Using this system, several mechanisms for the toxicity of extracellular Aβ oligomers have been proposed. Aβ oligomers are shown to bind multiple cell surface receptors, including N-methyl-D-aspartate receptor (NMDAR), insulin receptor, cellular prion protein, and others, and disturb their normal function (reviewed in [8,37,38,39]). Aβ oligomers also bind the plasma membrane directly to form ion channel-like amyloid pores and disrupt cellular calcium homeostasis [37]. These mechanisms account for Aβ oligomer-induced synaptic dysfunction and neurodegeneration and implicate therapeutic strategies for AD.

**Figure 4:**
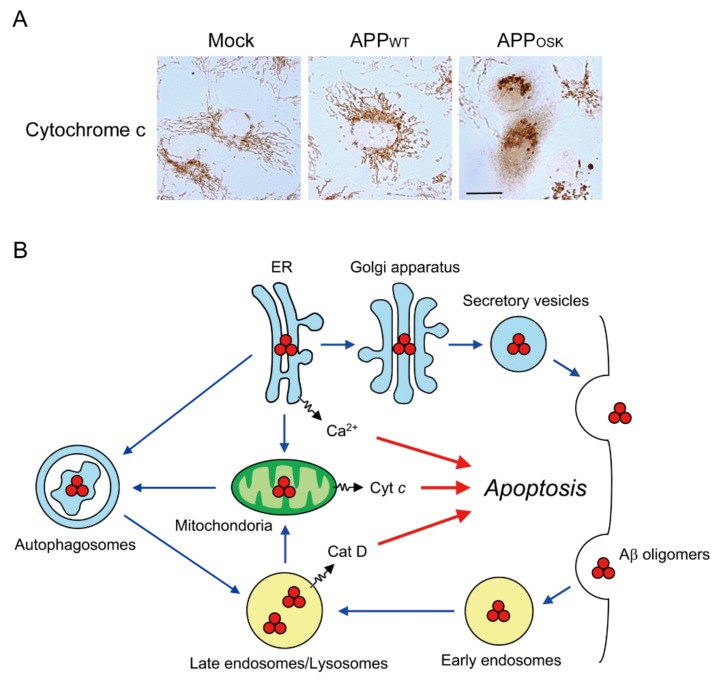
Cytotoxic effects of the Osaka mutation. (**A**) Disruption of mitochondria network by the Osaka mutation. Cytochrome c release from the mitochondria into the cytoplasm was detected in cells expressing the Osaka mutation. Scale bar = 20 μm. (**B**) Proposed mechanism underlying intracellular Aβ oligomer-induced cell death. Accumulation of Aβ oligomers caused ER stress, endosomal/lysosomal damage, and mitochondrial dysfunction, all of which eventually lead to apoptosis (red arrow). Blue arrows indicate the intracellular transport pathways of Aβ oligomers.

On the other hand, in the Osaka mutation, Aβ oligomers were detected predominantly within neurons. Additionally, their subcellular localization and influence on cellular functions had remained unclear. To address these questions, we generated cellular models by transfecting COS-7 cells with mutant APP. Cells were double stained with Aβ42 or Aβ oligomers and organelle markers for ER (calnexin), Golgi apparatus (furin), early endosomes (EEA1), late endosomes (M6PR), lysosomes (LAMP2), and autophagosomes (LC3). Intracellular Aβ42 was detected in all organelles tested with preferential localization in late endosomes and found to predominantly form oligomers [18]. It is known that misfolded proteins in the ER often cause ER stress and apoptosis. We found ER stress markers, such as the molecular chaperone Grp78 and phosphorylated (i.e., down-regulated) translation initiation factor eIF2a, and apoptosis markers, including cleaved caspase-3 and -4 and DNA fragmentation, which was detected by the TUNEL method, were all positive in mutant APP-transfected cells [18]. We also detected in these cells an increase of the E3 ubiquitin ligase HRD1, a marker of ER-associated degradation, which is another cellular response to ER stress, and the cell surface expression of annexin V, an indicator of early apoptosis, together with positive signals for propidium iodide, a probe for the loss of membrane integrity in late apoptosis and necrosis [40]. Furthermore, cells expressing mutant APP displayed endosomal/lysosomal membrane damage, which was demonstrated by the leakage of a pinocytic tracer, Lucifer yellow, and a lysosomal enzyme, cathepsin D, from these organelles into the cytoplasm [40]. This might be caused by Aβ oligomer-derived amyloid pore formation in the endosomal/lysosomal membrane. In addition to the above organelle, intracellular Aβ oligomers also localized into the mitochondria with a marker for Tom20, and caused mitochondrial dysfunction, which was indicated by the aberrant fluorescence of JC-1, a reporter dye of the mitochondrial membrane potential, and the release of cytochrome c from the mitochondria into the cytoplasm, which is a sign of mitochondria-dependent apoptosis [40] (Figure 4A). These results collectively indicate that intracellular Aβ oligomers cause ER stress, endosomal/lysosomal damage, and mitochondrial dysfunction, all of which eventually lead to apoptosis (Figure 4B).

Following these findings, we examined whether these pathological events actually occur in the neurons of the Tg mouse brain. In 18-month-old APP_OSK_ mice, ER stress, lysosomal leakage, mitochondrial dysfunction, and apoptosis were shown to correlate with Aβ oligomer accumulation [40]. Our finding that the Osaka mutation induces the intracellular accumulation of Aβ oligomers followed by ER stress was confirmed by others using iPS cells derived from a patient with the Osaka mutation and from one sporadic AD patient [41]. Based on their results, the authors proposed that AD could be classified into two categories: extracellular Aβ type and intracellular Aβ type [41]. This is an interesting idea. However, in the brain of patients and model mice, the intracellular accumulation of Aβ is always followed by its extracellular deposition and these two events do not occur independently [11,42,43,44,45,46,47]. In some cases of AD, including the Osaka mutation, the excretion of Aβ aggregates from cells into the extracellular space might be impaired, resulting in the prolonged intracellular Aβ accumulation.

The above findings may explain the mechanism by which intracellular Aβ oligomers cause cell death. However, synaptic dysfunction by intracellular Aβ occurs long before neurodegeneration in Tg mice. In vitro studies have shown that extracellular Aβ oligomers alter synaptic spine density and morphology and disrupt axonal transport [48,49,50,51,52] and that these toxic effects are mediated by cell surface receptors, such as NMDAR [49,50], an ephrine receptor EphA4 [39], and a Nogo receptor NgR1 [53]. On the other hand, the effects of intracellular Aβ oligomers on synaptic spines and axonal transport were unclear. Thus, we transfected mouse or rat primary neurons with mutant and wild-type human APP and compared their effects. Initially, we confirmed that Aβ oligomers accumulated in neurons expressing Osaka-mutant APP, but not in those expressing wild-type APP [54]. The Osaka mutation induced a significant decrease in dendritic spine density with a dominant decrease of mushroom-type mature spine [54]. This toxic effect was tau-independent and unlikely to be caused by extracellular Aβ. Furthermore, this mutation disrupted the axonal and dendritic transport of BDNF, which was also tau-independent, and mitochondria. Spine formation and growth requires membrane trafficking mediated by recycling endosomes. The Osaka mutation was shown to also inhibit dendritic transport of recycling endosomes. These results could account for the synaptic alterations in the Osaka mutation.

The secretion of Aβ from cells was remarkably suppressed by the presence of the Osaka mutation. This may occur because the protein quality control system in the ER recognizes Osaka-mutant Aβ oligomers as a misfolded protein and thereby inhibits their transport from the ER to the cell surface. This begs the question: Is Aβ secretion necessary for neural function? In other words, what is the physiological role of Aβ secretion? We had proposed that Aβ may play a role in cholesterol efflux from cells and from the brain [55]. During its secretion, Aβ assembled lipoprotein-like particles with cellular excess cholesterol, an assembly mediated by the ATP-binding cassette transporter A1. Soon after the secretion, the particles may be fused with apoE-containing nascent high-density lipoprotein (HDL), forming mature HDL. If this hypothesis is true, the Osaka mutation may have a defect in cholesterol efflux and thereby induce the intracellular accumulation of cholesterol. This would be unfavorable for the cells because, for example, cholesterol accumulation in mitochondria has been shown to increase the susceptibility of neurons to Aβ-induced reactive oxygen species (ROS) generation [56]. To test this possibility, we transfected COS-7 cells with wild-type and mutant APP and compared their cholesterol content after cholesterol loading. It is known that after its internalization, cholesterol is transported from the endosomes to the ER and Golgi apparatus, which is mediated by the endosomal cholesterol transporters Niemann–Pick type C1 (NPC1) and type C2 (NPC2) and cytosolic cholesterol transporters such as oxysterol-binding protein-related proteins [57]. Cholesterol is then transported to the plasma membrane and excreted from the cells as a lipoprotein. Compared with mock-transfected cells, wild-type APP-expressing cells exhibited an immediate clearance of cholesterol from the cells, whereas Osaka-mutant APP-expressing cells showed a prolonged accumulation of cholesterol [58]. In the Osaka mutation, intracellular cholesterol dominantly localized to the ER and less to the Golgi apparatus and endosomes. This subcellular localization was similar to that of Aβ, suggesting that intracellular cholesterol transport largely depends on Aβ trafficking. The impaired cholesterol transport observed in the Osaka mutation resembles the phenotype observed in cellular models of Niemann–Pick disease type C [59] which is caused by pathological mutations in NPC1 and/or NPC2 genes and shows neural dysfunction, including dementia. The Osaka mutation also caused an accumulation of Aβ and cholesterol in the mitochondria and increased ROS generation within the cells [58]. These results indicate that the cytotoxic effects of the Osaka mutation are, at least in part, displayed via an impaired ability to mediate cholesterol transport and efflux. This finding implies that high levels of cholesterol may aggravate the pathological phenotype of the Osaka mutation. Our finding that hypercholesterolemia accelerated the intraneuronal accumulation of Aβ oligomers and memory loss in APP_OSK_ mice [60] appears to support this notion.

## 4. Mechanism of Recessive Inheritance of the Osaka Mutation

As mentioned above, the Osaka mutation causes disease only in homozygotes, implying that it is recessive. When we found the Osaka mutation, all other APP mutations identified previously in familial AD were dominant (https://www.alzforum.org/mutations/app). Why is the Osaka mutation recessive even though it makes a high amount of toxic Aβ oligomers?

Whether a mutation shows a dominant or recessive inheritance is determined by one of the following four conditions. (1) The mutant protein acquires a new, often toxic, function (gain of function). (2) The mutant protein loses its function (loss of function) and simultaneously disturbs the action of its normal counterpart (dominant negative effect). (3) The mutant protein loses its function and the amount of normal counterpart (only half the usual level) is not enough to maintain physiological function (haploinsufficiency). In cases 1–3, pathological phenotypes appear even in heterozygotes, and the mutation is recognized as dominant. Almost all APP mutations cause gain-of-toxic function, increasing Aβ (particularly Aβ42) production and/or enhancing Aβ aggregation into fibrils, and thus are classified as case 1. Finally, in the last condition, (4) the mutant protein loses its function, but the normal counterpart can maintain physiological function even at half the usual level. In this case, pathological phenotypes can be observed only in homozygotes, and the mutation is regarded as recessive.

According to this rule, the Osaka mutation was assumed to cause a loss of certain APP function. The next question we asked was which function of APP is lost by the mutation? APP has been proposed to have several functions as a receptor, cell adhesion molecule, and growth factor [61,62,63], but the exact physiological role of APP is not well known. Thus, we created a new mouse model harboring the Osaka mutation. Using the homologous recombination technique, the codon CAA was deleted in endogenous mouse APP exon 17 to knock-in the Osaka mutation. Different from APP_OSK_ mice, this knock-in mouse model, named OSK-KI mice, produces mouse Aβ under the original mouse APP promotor [64]. In 2004, homozygous KI mice were established. This model is considered suitable for studying the influence of the mutation on APP function, because it lacks wild-type APP, which may compensate for the function lost in mutant APP, and avoids the artifacts by APP overexpression that is often observed in Tg mice.

We compared the memory and Aβ oligomer pathology between homo-, hetero-, and non-KI mice at 4, 6, and 8 months by the Morris water maze and immunohistochemical and biochemical analyses. Only homo-KI mice displayed memory impairment and intraneuronal accumulation of Aβ oligomers followed by synapse loss, tau hyperphosphorylation, glial activation, and neuron loss [64]. This result represents the recessive inheritance of the Osaka mutation and furthermore demonstrates that this mutation promotes Aβ oligomerization even in mouse Aβ. However, unlike Tg mice, the timing of the Aβ oligomer accumulation and of the memory impairment do not match in KI mice; the former was observed at 8 months and the latter at 4 months. In contrast, in APP_OSK_ mice, memory impairment was detected soon after the intraneuronal accumulation of Aβ oligomers.

We then examined synaptic plasticity by electrophysiology in hippocampal slices prepared at 4 and 8 months. HFS was delivered to the molecular layer of the dentate gyrus, and fEPSP was recorded in the same region. In the presence of a GABA_A_ receptor antagonist, picrotoxin, HFS induced LTP in all groups (homo-, hetero-, and non-KI mice) at 4 months [64]. However, at 8 months, LTP was elicited only in the hetero- and non-KI mice. The LTP impairment in homo-KI mice was probably caused by the intraneuronal accumulation of Aβ oligomers. Interestingly, when the experiment was carried out without picrotoxin, something strange happened. Under the picrotoxin-free condition, GABAergic inhibitory inputs sufficiently prevented neuronal activation and thereby disturbed LTP induction as seen in hetero- and non-KI mice. On the contrary, in homo-KI mice, LTP was abnormally induced at 4 months, although it had diminished by 8 months again. These results suggest that in homo-KI mice, GABAergic neurotransmission is attenuated. Reduced inhibitory signaling would result in abnormal neural activation to impair normal cognition.

**Figure 5:**
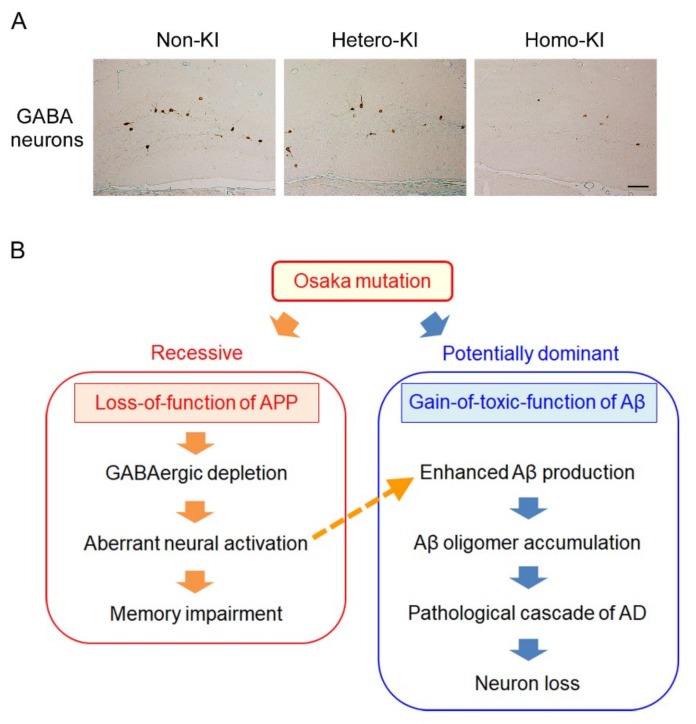
Phenotypes of OSK-KI mice. (**A**) GABAergic depletion by the Osaka mutation. Parvalbumin-positive GABA neurons in the dentate gyrus were decreased only in homo-KI mice. Scale bar = 30 μm. (**B**) Proposed mechanism for the recessive inheritance of the Osaka mutation. The Osaka mutation has dual effects; a loss-of-function of APP and gain-of-toxic-function of Aβ. The former causes GABAergic depletion, leading to abnormal neural activation and impaired cognition. The extensive neural activation results in enhanced Aβ production (dashed arrow). Then, the latter effect accelerates Aβ oligomerization that initiates the pathological cascade of AD leading to neuron loss.

While we observed this phenomenon in 2014, we came across a paper showing that APP is highly expressed in GABA interneurons in the dentate gyrus and plays an essential role in GABAergic synapse formation [65]. This information led us to speculate that the Osaka mutation spoils APP function for the maintenance of dentate GABA neurons. To test this hypothesis, we counted the number of parvalbumin-positive GABA neurons in the dentate gyrus of OSK-KI mice. As we expected, compared with non-KI mice, only homo-KI mice showed a decrease of GABA neurons at 4 months [64] (Figure 5A). It has been shown that Aβ production depends on neuronal activity [66,67] and that the secreted Aβ, in turn, regulates synaptic activity [68]. Insufficient GABAergic inputs will lead to enhanced Aβ production via an abnormal activation of neurons. This may cause an eventual accumulation of Aβ oligomers when Aβ has an oligomer-prone mutation, as seen in homo-KI mice at 8 months. If this is the case, early treatment of homo-KI mice with a GABAergic supplement could normalize cognition and prevent Aβ oligomer accumulation. Thus, to activate GABAergic neurotransmission, we orally administered diazepam, a positive allosteric modulator of GABA_A_ receptor, to 6-month-old homo-KI mice for 2 months and examined their memory and Aβ oligomers at 8 months. While the number of GABA neurons in the dentate gyrus remained lower, cognitive function was recovered and Aβ oligomers were not detected in the treated mice [64].

Taken together, our findings indicate that the Osaka mutation primarily causes a loss of APP function that is essential for GABA neurons (Figure 5B). The resultant imbalance between excitatory and inhibitory inputs will lead to impaired cognition [69] and also enhanced Aβ production via abnormal neural activation. In the presence of the Osaka mutation, Aβ promptly forms oligomers and accumulates within neurons, which initiates the pathological cascade of AD. Thus, in patients and KI mice with this mutation, Aβ oligomer accumulation can be regarded as a secondary phenomenon that follows the loss of APP function. This is the mechanism we propose to account for the recessive inheritance of the Osaka mutation. In APP_OSK_ mice, mutant Aβ is produced under the powerful mouse prion protein promotor, and therefore extensive neural activation is not necessary for Aβ oligomer accumulation.

As described above, early treatment with diazepam, which is an antiepileptic drug, prevented disease onset in OSK-KI mice. Epileptic seizures, which are caused by excessive neural activation, are frequently observed in the early stages of AD and are likely responsible, in part, for the progression of AD [70]. A recent study has shown that extracellular Aβ oligomers mediate neuronal hyperactivation through blocking glutamate reuptake by astrocytes [71]. This mechanism implies the existence of a vicious cycle between Aβ production and neuronal activation. In AD mouse models, certain classes of antiepileptic drugs exhibited disease-modifying properties [72,73,74]. These findings suggest a new therapeutic strategy for AD targeting neural network hyperexcitability.

## 5. Concluding Remarks

The Osaka mutation is the first recessive mutation found in familial AD. Interestingly, it has dual effects: a loss-of-function of APP and a gain-of-toxic-function of Aβ. The former causes GABAergic depletion, leading to abnormal neural activation and impaired cognition. The latter causes accelerated Aβ oligomerization that initiates the pathological cascade of AD including tau hyperphosphorylation, synapse loss, glial activation, NFT formation, and eventual neuron loss, although the effect never appears until enhanced Aβ production is established by the former effect. The most striking feature of the Osaka mutation is no fibrillization of Aβ. Our finding that AD develops only by Aβ oligomers without amyloid plaque formation strongly supports the hypothesis that Aβ oligomers play a critical role in the onset and progression of AD [1]. Furthermore, it raises an important question on the definition of AD: Is the existence of amyloid plaques an absolute requirement for AD? We propose that AD should be redefined as a neurodegenerative dementia caused by Aβ accumulation regardless of the Aβ aggregation state.

In the Osaka mutation, Aβ oligomers accumulate within neurons, localizing into the ER, Golgi apparatus, endosomes, lysosomes, autophagosomes, and mitochondria. This accumulation disrupts the axonal and dendritic transport of mitochondria, BDNF, and recycling endosomes, resulting in synaptic alterations. It also causes ER stress, endosomal/lysosomal leakage, and mitochondrial dysfunction, which lead to cell death.

Finally, the Osaka mutation provides a useful means to investigate the pathological and physiological roles of Aβ oligomers without the influence of amyloid fibrils or plaques (for example, [75,76,77,78]). Additionally, our model mice harboring this mutation could be a good tool for developing effective approaches in the prevention, treatment, and diagnosis of AD that specifically target Aβ oligomers (for example, [79,80]).
